# Clinical Society of Maryland

**Published:** 1887-04

**Authors:** 


### Society Reports.

### THE CLINICAL SOCIETY OF MARYLAND.

[Official Report.]

Stated Meeting, held February 4, 1887.

The President, Randolph Winslow, M. D., in the chair.

Dr. J. H. Branham read the notes on a case of

### CHRONIC CERVICAL ABSCESS.

Thos. Anderson, white, æt. 21 years. Born in Baltimore; father killed; mother died from causes not known to the writer. Three sisters, all enjoying good health. Five years ago the patient suffered from disease of the fifth metatarsal bone on the left side. This resulted from an injury. After removal of the dead bone the wound healed rapidly. About three years ago the patient had a fall and struck upon his left mastoid process, which resulted in much local swelling, pain and more or less discharge from the ear. About a year later a large abscess opened in the side of the neck, and has been discharging ever since. On 25th of June last the opening was enlarged and the abscess cavity explored. A small piece of exfoliated bone was found in the most dependent part of the suppurating tissues. There was no bare bone found, but this piece must have come from the mastoid process, which was partly gone. The original seat of disease had been relieved by nature, and this exfoliated bit of necrosed bone had kept up the trouble. The cavity is rapidly filling.

The doctor does not consider the process of tuberculous origin.

Dr. Frank Donaldson, Jr., reported a case of

### HÆMO-THORAX.

He was called in consultation to see a boy who had received a stab wound in the left thorax, the knife entering just below the fourth rib and penetrating to a distance of about three inches.

Physical exploration of the chest revealed the presence of much effusion and some tympanicity. On the following day he was called to see the patient in great haste. He found him in a rather serious condition. The chest was about two-thirds filled with fluid. He aspirated and drew off 26 ounces of bloody serum. There was no return of the hæmorrhage and the boy got well. He thought it probable that the hæmorrhage from the lung was checked by the expansion of the lung that followed removal of the fluid.

### DISCUSSION.

Dr. Randolph Winslow thinks the position of the wound, as indicated by Dr. Donaldson, would lead one to suspect injury to the intercostal artery.

Dr. Donaldson could not say that the artery was not injured, but he certainly thought the knife had penetrated sufficiently far to injure the lung tissue. The boy is said to have expectorated blood before he saw him. He saw no expectoration of blood after he was called in.

Dr. Henry Rolando related a case of

### STAB-WOUND IN THE CHEST.

The thorax was punctured below the left clavicle in the first intercostal space. Hæmorrhage was immediate and continued until the left pleural sac was about half filled with blood. The man was kept quiet. After several days, the amount not having increased, the chest was aspirated. The operation was repeated three or four times, each time bringing off a considerable quantity of blood and serum. The patient recovered.

Dr. Winslow thinks in Dr. Donaldson's case there was injury to an intercostal artery, and likewise a pleuritic effusion. He thinks the course pursued by Dr. Donaldson the proper one.

Dr. Randolph Winslow read the following case:

### TUBERCULOSIS OF SOFT PARTS OF THIGH SIMULATING BONE DISEASE.

Collins, a young white man, was taken with pain and swelling of thigh in December, 1884, followed by an abscess which was opened and drained by Dr. Michael. His health improved, but a sinus remained, opening at middle and outer part of thigh, and pain about trochanter, and frequently an erysipelatous blush. Discharge neither free nor offensive. Supposing the man to be suffering with central osteitis, an incision was made by Dr. Winslow down to the bone, but no necrosis or caries could be detected. Three months later, cut down on trochanter, and thoroughly scraped abscess cavities, but could not discover any disease of the bone. The man was run down, said he had syphilis and had scars in the groin. From Dr. Winslow he fell into Dr. Tiffany's service, and eventually was sent to Bay View, where he died in November, 1886. The chief interest centers in the post-mortem examination. The liver was much enlarged and was amyloid, kidneys inflamed and amyloid, spleen enlarged. Intestines and rectum ulcerated and covered with membrane. No spinal caries, no osteomyetitis, some periostitis, soft parts of thigh showed tuberculosis and were the seat of the changes which finally caused his death. The result of the autopsy was a great surprise, as it was believed that there was disease of the femur.

DISCUSSION.

Dr. O. J. Coskery thinks that in all these cases there are certain diatheses that should be considered in connection with the treatment; for example, the syphilitic, the gouty, the rheumatic, tuberculous, etc. He referred to a case in which there was every evidence of trouble with the femur, but in which there was so very much œdema of the surrounding soft tissues that satisfactory exploration of the bone was impracticable. He suspected the man of being gouty and placed him upon anti-gout treatment. In three weeks from the commencement of this treatment, the man was going about attending to his daily occupation. He had not been able to do work of any kind for three months before.

Dr. John Chambers thinks in cases such as are related by Dr. Winslow, there is usually such a wide-spread burrowing of pus that the field for infection is in consequence very large. He is always inclined to doubt, therefore, whether the diseased condition results from the start from the presence of tubercle bacilli or whether the bacilli are secondary, having taken up their abode in this favorable media.

Dr. J. Edwin Michael related a case of

### RESECTION OF THE LOWER JAW.

It occurred in a man, æt. 54 years, in whom there was a carcinomatous process involving the floor of the mouth, the anterior end of the tongue and the anterior portion of the body of the bone on each side of the median line.

The case was fully explained to the patient, and with his consent the operation was done as holding out the only chance for relief.

The operation consisted in an incision in the median line of the lower lip down to the lower edge of the bone, and then around the bone on either side in order to sever its attachments to the cheek muscles. The bone was then sawed through on either side with the chain-saw at the point from which had been extracted the second bicuspid teeth. The knife was then passed through the neck in the median line just above the hyoid bone and up through the base of the tongue. Incisions were then made from the point of puncture to the right and left, terminating at the point of section of the bone. In this way a V-shaped opening was left in the stump of the tongue, the apex of the V pointing toward the epiglottis. After separating the remaining attachments the tissues were held in position by the following device: The sides of the V in the tongue were brought together in the median line and the stump thus left was attached by silver wire to a wire running transversely across the mouth and connecting together the remaining portions of the bone. The tongue was thus prevented from gravitating into the pharynx.

The hyoid bone was supported by a second wire running from it to the same tranverse wire. Drainage was obtained through the opening in the neck. The wound was dressed with iodoform, gauze and cotton. The dressing extended from the incision in the lip down to and covering the wound in the neck.

On the second day the patient drank fluid and communicated by writing that he was moderately comfortable under the circumstances.

After the second day the outlook became less favorable, and on the eighth day the patient died of septicæmia.

While Dr. Michael thinks operation holds out the only chances for recovery in these cases, yet the liability to recurrence and the difficulty of antiseptic practice in this region are so great that he never operates without stating these points plainly to the patient.

Experience has shown these cases to turn out disastrous to the patient in so many instances that he always operates with some feeling of doubt.

### EPITHELIOMA OF THE TONSIL.

Dr. F. Donaldson, Jr., referred to nine reported cases of epithelioma of the tonsil, in four of which operation resulted in recovery.

Dr. J. E. Michael then reported

### AN AMPUTATION IN THE UPPER THIRD OF THE THIGH FOR OSTEO-SARCOMA.

The history of this case is as follows: During last July a swelling appeared above the knee. Its nature was not recognized by the physician in attendance and Dr. Tiffany was called in. He diagnosed osteo-sarcoma of the periosteal variety and recommended amputation. The patient objected to the operation and procured the services of a charlatan who, by means of a certain mud applied locally, succeeded, much to the delight of the patient, in greatly reducing the surrounding œdema, though no reduction whatever took place in the actual size of the tumor. This mud-doctor then called in a friend, who aspirated from the growth a small amount of bloody fluid. He was desirous of incising the growth, but was refused. The case then came into the hands of Dr. Michael, who confirmed the diagnosis made by Dr. Tiffany, and recommended high amputation through the thigh. He recommended amputation through the thigh in preference at the hip-joint because of the age of the patient. The latter operation would have been preferred had the man been younger. The ordinary flap amputation was made under the most detailed antiseptic precautions. There was considerable shock after the operation, but from this he readily rallied under the use of stimulants. On the fourth day the dressing dropped off and the stump was again dressed by the student in charge with iodoform and oakum. The dressing again came off on the sixth day. On the seventh day the patient became delirious and the pulse and temperature went up. Iodoform poisoning was suspected and the dressings were removed, the stitches cut and the surface freed from all iodoform and dressed with carbolized lint, after which the symptoms rapidly disappeared. Dr. Michael thinks the success of these operations depends, in a great measure, upon the perfection of the antiseptic methods employed.

DISCUSSION.

Dr. O. J. Coskery is of the opinion that a history of injury to the femur, followed by swelling of a peculiar consistency and appearance, should remove all difficulty in diagnosis. He referred to three cases of osteo-sarcoma of the femur following upon injury. He thinks tumors of the femur following upon injury are, as a rule, of osteo-sarcomatous nature.

Dr. J. E. Michael, in answer to Dr. Branham, said he diagnosed iodoform poisoning on the following points: Delirium, rapidity of pulse, elevation of temperature, which, however, is not constant, and the disappearance of the symptoms when the drug is withdrawn.

Dr. John Chambers thinks the trouble was not iodoform poisoning, but rather of septic origin—the infection having taken place when the dressings were removed.

Dr. J. E. Michael thinks the sudden appearance of the symptoms and their equally sudden disappearance rather at variance with any septic phenomenon with which he is acquainted.

Dr. W. E. Moseley, in speaking of the sudden alterations in temperature in obstetrical cases, considers them more of nervous than of septic origin.

He called attention to certain idiosyncrasies possessed by some patients toward iodoform. He has patients in whom severe headache is always a consequence of the use of iodoform.

Dr. J. E. Michael has observed these peculiarities also.

